# Risk Factors for Chronic Disease in Viet Nam: A Review of the Literature

**DOI:** 10.5888/pcd10.120067

**Published:** 2013-01-10

**Authors:** Damian Hoy, Chalapati Rao, Nguyen Thi Trang Nhung, Geoffrey Marks, Nguyen Phuong Hoa

**Affiliations:** Author Affiliations: Chalapati Rao, Geoffrey Marks, School of Population Health, University of Queensland, Queensland, Australia; Nguyen Thi Trang Nhung, Nguyen Phuong Hoa, Hanoi School of Public Health, Ho Chi Minh City, Viet Nam.

## Abstract

**Introduction:**

Chronic diseases account for most of the disease burden in low- and middle-income countries, particularly those in Asia. We reviewed literature on chronic disease risk factors in Viet Nam to identify patterns and data gaps.

**Methods:**

All population-based studies published from 2000 to 2012 that reported chronic disease risk factors were considered. We used standard chronic disease terminology to search PubMed and assessed titles, abstracts, and articles for eligibility for inclusion. We summarized relevant study information in tables listing available studies, risk factors measured, and the prevalence of these risk factors.

**Results:**

We identified 23 studies conducted before 2010. The most common age range studied was 25 to 64 years. Sample sizes varied, and sample frames were national in 5 studies. A combination of behavioral, physical, and biological risk factors was studied. Being overweight or obese was the most common risk factor studied (n = 14), followed by high blood pressure (n = 11) and tobacco use (n = 10). Tobacco and alcohol use were high among men, and tobacco use may be increasing among Vietnamese women. High blood pressure is common; however, people’s knowledge that they have high blood pressure may be low. A high proportion of diets do not meet international criteria for fruit and vegetable consumption. Prevalence of overweight and obesity is increasing. None of the studies evaluated measured dietary patterns or total caloric intake, and only 1 study measured dietary salt intake.

**Conclusion:**

Risk factors for chronic diseases are common in Viet Nam; however, more recent and context-specific information is required for planning and monitoring interventions to reduce risk factors and chronic disease in this country.

## Introduction

### The burden of chronic diseases

Chronic diseases, particularly stroke, heart disease, hypertension, diabetes, cancer, chronic lung disease, and musculoskeletal disorders, account for most disease burden in low- and middle-income countries, particularly those in Asia (1–3). Chronic diseases caused an estimated 36 million deaths worldwide in 2008, which represented more than 63% of all global deaths. Ninety percent of these deaths occurred in low- and middle-income countries (4).

In addition to the effect of chronic diseases on mortality and morbidity, these diseases also present macroeconomic and developmental challenges. Chronic diseases affect the most productive years of life. At the household level, they cause loss of productivity and income from disability and death and can further compound the extent of poverty because of the high cost of health care. The World Bank estimates that, in India, between 4% and 10% of the potential gross domestic product is foregone each year because of chronic diseases. The World Health Organization (WHO) estimates that, in China, lost productivity from chronic diseases may cost US $550 billion between 2005 and 2015 (5).

Recent national assessments of mortality and causes of death in Viet Nam have identified stroke as the leading cause of death in both men and women (6). These data were used in the 2008 Viet Nam Burden of Disease and Injury Study, which found that chronic diseases were responsible for 66% of the overall disease burden in men and 77% in women (7). Although this magnitude of chronic disease burden was similar to that of developed countries (8), the magnitude of burden from stroke was substantially higher in Viet Nam, where stroke caused the greatest burden of all diseases and injuries in 2008. In men and women aged 45 to 69 years, stroke caused 14% and 9% of the overall burden, respectively. In people aged 70 years or older, stroke caused 22% of the burden in males and 24% in females (7). Lung cancer, chronic obstructive pulmonary disease (COPD), diabetes, liver disease, and osteoarthritis were among the other major causes of disease burden in 2008. Effective strategies to reduce risk factors for chronic disease are required to address these health concerns in Viet Nam.

### Risk factors of chronic diseases

The most common chronic diseases share risk factors (5), which are often classified as behavioral or biological. The main modifiable behavioral risk factors are tobacco use, alcohol use, an unhealthful diet, and physical inactivity (9). The main biological risk factors are overweight, obesity, high blood pressure, elevated blood glucose, and abnormal blood lipids and its subset, raised total cholesterol (9).

These risk factors are responsible for most of the burden of death and disability throughout the world, regardless of a country’s economic status (10). However, the exposure that individuals and populations have to these risk factors is much higher in low- and middle-income countries than in high-income countries, where comprehensive interventions are in place to help protect people (5). Risk factors for chronic diseases in low- and middle-income countries are especially common among disadvantaged groups (5).

### Chronic disease risk factors in Viet Nam

In Viet Nam, the main risk factors for chronic diseases include tobacco use, alcohol use, and local dietary patterns including the preferential consumption of foods that are high in salt and in saturated and partially hydrogenated fats (5). To understand the risk factors underlying the growing burden of chronic diseases in Viet Nam, several surveys have been conducted during the past 10 years. The information collected is crucial for the development of context-specific and culturally appropriate policy and interventions for the prevention and management of chronic diseases. The objective of our study was to assess the extent of chronic disease risk factors in Vietnamese adults and to identify information gaps. To meet this objective, we reviewed the literature from 2000 through 2012.

## Methods

### Selection criteria

All population-based studies published from January 1, 2000, to August 19, 2012, that reported on chronic disease risk factors in Viet Nam were considered for inclusion. The year 2000 was chosen to coincide with the introduction of the WHO STEPwise Approach to Surveillance (STEPS) chronic disease surveillance program. Studies were excluded if 1) they had a sample size of fewer than 1,000, 2) they were not conducted in Viet Nam, 3) they were conducted before 2000, 4) they were a review article (did not report original research), 5) they were not population-based, 6) they were limited to a subset of the population (eg, pregnant women), 7) they were limited to children or adolescents, or 8) their results had been presented in another article included in our review.

### Search strategy

The search strategy was consistent with a systematic review of risk factor information in India (11). We used the following keywords in combination with “Viet*” AND “epidemiology OR prevalence OR distribution” to search the PubMed electronic database: tobacco, alcohol intake, fruit intake, vegetable intake, physical activity, exercise, sedentary lifestyle, BMI, overweight, obesity, waist circumference, waist hip ratio, blood pressure, hypertension, metabolic syndrome, diabetes, blood sugar, hyperglycaemia, dysglycaemia, glucose abnormalities, cholesterol, lipids, coronary heart disease, myocardial infarction, angina, heart, coronary, cardiovascular, ischaemic heart disease, stroke.

There were no sex or language restrictions. We inspected reference lists of included studies to identify additional relevant studies. One researcher (D.H.) assessed the titles and abstracts of all retrieved references to identify studies that appeared to fulfill inclusion criteria, and we retrieved all potentially eligible articles in full text. We also contacted relevant authorities in Viet Nam to seek unpublished information on chronic disease risk factor prevalence.

### Data extraction and management

One researcher (D.H.) extracted relevant study information and input it into a Microsoft Excel 2007 database (12). Variables extracted included citation, year of study, study age range, sex, sample size, coverage, risk factor measured, measurement method, threshold, and result.

## Results

### Search results

The electronic database search yielded 506 articles (Figure). Of these, we excluded 466 titles that met our exclusion criteria, which left 40 eligible titles. Of these, we excluded 11 abstracts that met our exclusion criteria, leaving 29 eligible abstracts. An additional 3 papers were identified from inspection of the reference lists of relevant articles.

**Figure:**
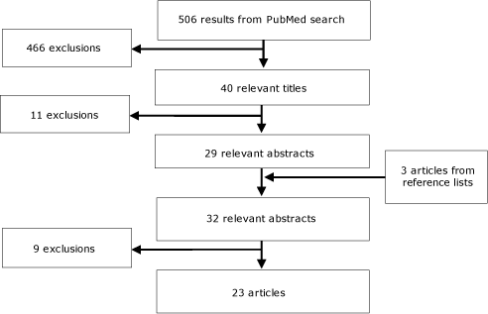
Search strategy and exclusion process for literature review of articles related to risk factors for chronic disease in Viet Nam. Articles were excluded if they met the following 4 exclusion criteria: 1) they had a sample size less than 1000 (n = 8), 2) they were limited to a subset of the population (eg, pregnant women) (n = 4), 3) they were limited to children or adolescents (n = 2), or 4) they had already presented their results in another paper included in the review (n=6). A text version of this figure is available.

The full-text articles of the 32 relevant abstracts were assessed and 9 were excluded because they met 1 or more of the following 4 original exclusion criteria: 1) they had a sample size less than 1,000 (n = 8), 2) they were limited to a subset of the population (eg, pregnant women) (n = 4), 3) they were limited to children or adolescents (n = 2), or 4) they had already presented their results in another paper included in the review (n = 6). Twenty-three studies were eligible (Figure).

### Description of included studies

All 23 studies were conducted before 2010; 2 were conducted in 2009, 1 in 2008, and the rest from 2000 through 2005. The most common age range studied was 25 to 64 years (13 of the 23 studies), which corresponds with the WHO STEPS approach (10). Sample sizes varied from 643 to 158,019.

The sampling frame was national for 5 studies and 2 provinces for 1 study. Of those remaining, the sample frame was Ho Chi Minh City for 5 studies, Fila Bavi district (rural) for 3 studies, Fila Bavi and Chililab districts (rural) for 6 studies, an unnamed rural district for 2 studies and Can Tho (urban and rural) in Southern Viet Nam for 1 study (Table).

**Table:** Risk Factors for Chronic Disease in Viet Nam: 23 Articles Reviewed and Summary of Data Reported

Topic/Citation	Year(s) of Study		Population Age, y	Sample Size	Georgraphic Area Covered	Measurement Method	Definition^a^	Prevalence
**Abdominal adiposity, male**								
Trinh et al 2010 (28)	2005		25–64	908	HCMC	Waist circumference (from midpoint of last palpable rib and top of hip bone)	≥90 cm	11.3%
Cuong et al 2007 (29)	2004		20–60	717	HCMC	Waist circumference (at navel level)	≥79 cm	30.0%
Son et al 2012 (30)	2002–2008		≥25	3,853	National	Waist circumference (from midpoint of last palpable rib and top of hip bone)	≥90 cm	3.9%
Trinh et al 2009 (31)	2005		25–64	908	HCMC	Waist circumference (from midpoint of last palpable rib and top of hip bone)	≥94 cm	5.9%
**Abdominal adiposity, female**								
Trinh et al 2010 (28)	2005		25–64	1,063	HCMC	Waist circumference (from midpoint of last palpable rib and top of hip bone)	≥80 cm	24.6%
Cuong et al 2007 (29)	2004		20–60	771	HCMC	Waist circumference (at navel level)	≥77 cm	27.0%
Son et al 2012 (30)	2002–2008		≥25	5,970	National	Waist circumference (from midpoint of last palpable rib and top of hip bone)	≥80 cm	19.7%
**Alcohol use, male**								
Trinh et al 2010 (28)	2005		25–64	908	HCMC	Questionnaire (self-report)	≥4 drinks/d or 9–12 drinks on any d	9.1%
Huu Bich et al 2009 (32)	2005		25–64	4,194	Fila Bavi district	Questionnaire (self-report)	≥5 drinks/d in last wk	31.0%
Huu Bich et al 2009 (32)	2005		25–64	4,194	Chililab district	Questionnaire (self-report)	≥5 drinks/d in last wk	17.0%
Pham et al 2009 (33)	2005		25–64	910	Can Tho (southern VN)	Questionnaire (self-report)	≥5 drinks on any d in last wk	39.0%
Van Minh et al 2008 (34)	2005		25–74	1,216	Fila Bavi district	Questionnaire (self-report)	≥2 standard drinks/d	61.0%
Giang et al 2008 (35)	2004		18–60	1,695	1 rural district	Questionnaire (self-report)	At-risk drinker (AUDIT >7)	25.5%
Nguyen et al 2012 (36)	2009		≥25	785	2 provinces	Questionnaire (self-report)	≥4 standard drinks/d	27.6%
**Alcohol use, female**								
Trinh et al 2010 (28)	2005		25–64	1,063	HCMC	Questionnaire (self-report)	≥3 drinks/d	0.4%
Huu Bich et al 2009 (32)	2005		25–64	4,194	Fila Bavi district	Questionnaire (self-report)	≥4 drinks/d in last wk	0.4%
Huu Bich et al 2009 (32)	2005		25–64	4,194	Chililab district	Questionnaire (self-report)	≥4 drinks/d in last wk	0.3%
Pham et al 2009 (33)	2005		25–64	1,066	Can Tho (southern VN)	Questionnaire (self-report)	≥5 drinks on any d in last wk	0.4%
Van Minh et al 2008 (34)	2005		25–74	1,268	Fila Bavi district	Questionnaire (self-report)	≥3 standard drinks/d	5.0%
Giang et al 2008 (35)	2004		18–60	1,728	1 rural district	Questionnaire (self-report)	At-risk drinker (AUDIT >5)	0.7%
Nguyen et al 2012 (36)	2009		≥25	1,345	2 provinces	Questionnaire (self-report)	≥3 standard drinks/d	0.9%
**Elevated blood cholesterol, male**								
Nguyen et al 2012 (36)	2009		≥25	785	2 provinces	Questionnaire (self-report) and blood sample after overnight fast	Dislipidaemia^b^	62.8%
**Elevated blood cholesterol, female**								
Nguyen et al 2012 (36)	2009		≥25	1,345	2 provinces	Questionnaire (self-report) and blood sample after overnight fast	Dislipidaemia^b^	52.4%
**Elevated total cholesterol, male**								
Trinh et al 2010 (28)	2005		25–64	908	HCMC	Capillary blood sample after 8–10 h fast	≥5.2 mmol/L	16.0%
Pham et al 2009 (33)	2005		25–64	910	Can Tho (southern VN)	Capillary blood sample after 8–10 h fast	≥5.2 mmol/L	14.5%
**Elevated total cholesterol, female**								
Trinh et al 2010 (28)	2005		25–64	1,063	HCMC	Capillary blood sample after 8–10 h fast	≥5.2 mmol/L	17.0%
Pham et al 2009 (33)	2005		25–64	1,066	Can Tho (southern VN)	Capillary blood sample after 8–10 h fast	≥5.2 mmol/L	21.0%
**Elevated blood glucose, both sexes**								
Duc Son et al 2004 (37)	2001		15+	2,932	HCMC	Venous blood sample from forearm after overnight fast	≥6.1 mmol/L	9.8%
**Elevated blood glucose, male**								
Trinh et al 2010 (28)	2005		25–64	908	HCMC	Capillary blood sample after 8–10 h fast	≥5.6 mmol/L	7.3%
Pham et al 2009 (33)	2005		25–64	910	Can Tho (southern VN)	Capillary blood sample after 8–10 h fast	≥6.1 mmol/L	1.0%
Cuong et al 2007 (29)	2004		20–60	717	HCMC	Venous blood sample from forearm after overnight fast	≥6.1 mmol/L	4.2%
Nguyen et al 2012 (36)	2009		≥25	785	2 provinces	Questionnaire; blood sample after overnight fast and 2 hours after OGTT	Diabetes^c^	8.0%
**Elevated blood glucose, female**								
Trinh et al 2010 (28)	2005		25–64	1,063	HCMC	Capillary blood sample after 8–10 h fast	≥5.6 mmol/L	5.6%
Pham et al 2009 (33)	2005		25–64	1,066	Can Tho (southern VN)	Capillary blood sample after 8–10 h fast	≥6.1 mmol/L	1.1%
Cuong et al 2007 (29)	2004		20–60	771	HCMC	Venous blood sample from forearm after overnight fast	≥6.1 mmol/L	4.3%
Nguyen et al 2012 (36)	2009		≥25	1,345	2 provinces	Questionnaire; blood sample after overnight fast and 2 hours after OGTT	Diabetes^c^	6.2%
**High blood pressure, both sexes**								
Duc Son et al 2004 (37)	2001		15+	2,932	HCMC	Calibrated mercury sphygmomanometer	≥140/90 mm Hg	26.2%
**High blood pressure, male**								
Trinh et al 2010 (28)	2005		25–64	908	HCMC	Digital automatic blood pressure monitor	≥130/85 mm Hg	38.0%
Van Minh et al 2009 (38)	2005		25–64	4,194	Fila Bavi district	Digital automatic blood pressure monitor	≥140/90 mm Hg	20.0%
Van Minh et al 2009 (38)	2005		25–64	4,194	Chililab district	Digital automatic blood pressure monitor	≥140/90 mm Hg	22.0%
Pham et al 2009 (33)	2005		25–64	910	Can Tho (southern VN)	Digital automatic blood pressure monitor	≥140/90 mm Hg	27.0%
Van Minh et al 2008 (13)	2005		25–64	993	Fila Bavi district	Questionnaire (self-report)	Lifetime prevalence	5.6%
Van Minh et al 2008 (13)	2005		25–64	1,096	Chililab district	Questionnaire (self-report)	Lifetime prevalence	7.1%
Van Minh et al 2007 (39)	2005		25–64	987	Fila Bavi district	Digital automatic blood pressure monitor	≥140/90 mm Hg	24.0%
Cuong et al 2007 (29)	2004		20–60	717	HCMC	Calibrated mercury sphygmomanometer	≥140/90 mm Hg	11.3%
Ng et al 2006 (40)	2002		25–64	997	Fila Bavi district	Digital automatic blood pressure monitor	≥140/90 mm Hg	18.0%
Nguyen et al 2012 (36)	2009		≥25	785	2 provinces	Digital automatic blood pressure monitor	≥140/90 mm Hg	31.2%
Son et al 2012 (30)	2002–2008		≥25	3,853	National	Digital automatic blood pressure monitor	≥140/90 mm Hg	28.3%
Tesfaye et al 2007 (41)	2003–2004		25–64	993	1 district	Digital automatic blood pressure monitor; questionnaire (self-report)	SBP ≥140mm Hg or DBP ≥90mm Hg or use of antihypertensive medication	19.3%
**High blood pressure, female**								
Trinh et al 2010 (28)	2005		25–64	1,063	HCMC	Digital automatic blood pressure monitor	≥130/85 mm Hg	21.0%
Van Minh et al 2009 (38)	2005		25–64	4,194	Fila Bavi district	Digital automatic blood pressure monitor	≥140/90 mm Hg	10.0%
Van Minh et al 2009 (38)	2005		25–64	4,194	Chililab district	Digital automatic blood pressure monitor	≥140/90 mm Hg	15.0%
Pham et al 2009 (33)	2005		25–64	1,066	Can Tho (southern VN)	Digital automatic blood pressure monitor	≥140/90 mm Hg	16.0%
Van Minh et al 2008 (13)	2005		25–64	1,023	Fila Bavi district	Questionnaire (self-report)	Lifetime prevalence	4.1%
Van Minh et al 2008 (13)	2005		25–64	1,108	Chililab district	Questionnaire (self-report)	Lifetime prevalence	5.5%
Van Minh et al 2007 (39)	2005		25–64	997	Fila Bavi district	Digital automatic blood pressure monitor	≥140/90 mm Hg	14.0%
Cuong et al 2007 (29)	2004		20–60	771	HCMC	Calibrated mercury sphygmomanometer	≥140/90 mm Hg	8.9%
Ng et al 2006 (40)	2002		25–64	999	Fila Bavi district	Digital automatic blood pressure monitor	≥140/90 mm Hg	10.0%
Nguyen et al 2012 (36)	2009		≥25	1,345	2 provinces	Digital automatic blood pressure monitor	≥140/90 mm Hg	25.0%
Son et al 2012 (30)	2002–2008		≥25	5,970	National	Digital automatic blood pressure monitor	≥140/90 mm Hg	23.1%
Tesfaye et al 2007 (41)	2003–2004		25–64	1,023	1 district	Digital automatic blood pressure monitor; questionnaire (self-report)	SBP ≥140mm Hg or DBP ≥90mm Hg or use of antihypertensive medication	9.4%
**High salt intake, male**								
Nguyen et al 2012 (36)	2009		≥25	785	2 provinces	Questionnaire (self-report)	Prefer daily foods containing more salt than similar foods ordered by others	32.2%
**High salt intake, female**								
Nguyen et al 2012 (36)	2009		≥25	1,345	2 provinces	Questionnaire (self-report)	Prefer daily foods containing more salt than similar foods ordered by others	27.1%
**Low fruit and vegetable consumption, male**								
Ahmed et al 2009 (42)	2005		25–64	4,194	Fila Bavi district	Questionnaire (self-report)	<5 servings of fruit and vegetables/d	87.0%
Ahmed et al 2009 (42)	2005		25–64	4,194	Chililab district	Questionnaire (self-report)	<5 servings of fruit and vegetables/d	64.0%
Pham et al 2009 (33)	2005		25–64	910	Can Tho (southern VN)	Questionnaire (self-report)	<5 servings of fruit and vegetables/d	70.0%
Nguyen et al 2012 (36)	2009		≥25	785	2 provinces	Questionnaire (self-report)	<5 servings of fruit and vegetables/d	59.4%
**Low fruit and vegetable consumption, female**								
Ahmed et al 2009 (42)	2005		25–64	4,194	Fila Bavi district	Questionnaire (self-report)	<5 servings of fruit and vegetables/d	88.0%
Ahmed et al 2009 (42)	2005		25–64	4,194	Chililab district	Questionnaire (self-report)	<5 servings of fruit and vegetables/d	58.0%
Pham et al 2009 (33)	2005		25–64	1,066	Can Tho (southern VN)	Questionnaire (self-report)	<5 servings of fruit and vegetables/d	74.0%
Nguyen et al 2012 (36)	2009		≥25	1,345	2 provinces	Questionnaire (self-report)	<5 servings of fruit and vegetables/d	52.0%
**Obesity, male**								
Razzaque et al 2009 (43)	2005		25–64	4,194	Fila Bavi district	BMI (height and weight measured using standard calibrated instruments)	≥30.0 kg/m^2^	0.1%
Razzaque et al 2009 (43)	2005		25–64	4,194	Chililab district	BMI (height and weight measured using standard calibrated instruments)	≥30.0 kg/m^2^	0.3%
Nguyen et al 2012 (36)	2009		≥25	785	2 provinces	BMI (height and weight measured using standard calibrated instruments)	BMI ≥25 kg/m^2^ or (BMI ≥ 23 kg/m^2^ with waist circumference ≥90 cm)	14.5%
**Obesity, female**								
Razzaque et al 2009 (43)	2005		25–64	4,194	Fila Bavi district	BMI (height and weight measured using standard calibrated instruments)	≥30.0 kg/m^2^	0.3%
Razzaque et al 2009 (43)	2005		25–64	4,194	Chililab district	BMI (height and weight measured using standard calibrated instruments)	≥30.0 kg/m^2^	0.3%
Nguyen et al 2012 (36)	2009		≥25	1,345	2 provinces	BMI (height and weight measured using standard calibrated instruments)	BMI ≥25 kg/m^2^ or (BMI ≥23 kg/m^2^ with waist circumference ≥80 cm)	17.4%
**Overweight or obesity, both sexes**								
Ha do et al 2011 (44)	2000		25–64	14,452	National	BMI (height and weight measured using standard calibrated instruments)	≥25.0 kg/m^2^	3.7%
Ha do et al 2011 (44)	2005		25–64	17,213	National	BMI (height and weight measured using standard calibrated instruments)	≥25.0 kg/m^2^	7.0%
Duc Son et al 2004 (37)	2001		≥15	2,932	HCMC	BMI (height and weight measured using standard calibrated instruments)	≥25.0 kg/m^2^	18.6%
**Overweight or obesity, male**								
Ta et al 2010 (45)	Not reported		30–70	643	HCMC	BMI (height and weight measured using standard calibrated instruments)	≥25.0 kg/m^2^	21.7%
Ahmed et al 2009 (42)	2005		25–64	4,194	Fila Bavi district	BMI (height and weight measured using standard calibrated instruments)	≥25.0 kg/m^2^	1.8%
Ahmed et al 2009 (42)	2005		25–64	4,194	Chililab district	BMI (height and weight measured using standard calibrated instruments)	≥25.0 kg/m^2^	6.7%
Pham et al 2009 (33)	2005		25–64	910	Can Tho (southern VN)	BMI (height and weight measured using standard calibrated instruments)	≥25.0 kg/ m^2^	11.1%
Tuan et al 2008 (46)	2002		18–65	158,019	National	BMI (height and weight measured using standard calibrated instruments)	≥25.0 kg/ m^2^	4.1%
Van Minh et al 2007 (39)	2005		25–64	987	Fila Bavi district	BMI (height and weight measured using standard calibrated instruments)	≥25.0 kg/m^2^	3.0%
Cuong et al 2007 (29)	2004		20–60	717	HCMC	BMI (height and weight measured using standard calibrated instruments)	≥23.0 kg/m^2^	32.0%
Nguyen et al 2007 (47)	2002		≥19	44,254	National	BMI (height and weight measured using standard calibrated instruments)	≥25.0 kg/m^2^	4.4%
Son et al 2012 (30)	2002–2008		≥25	3,853	National	BMI (height and weight measured using standard calibrated instruments)	≥23.0 kg/m^2^	18.4%
Tesfaye et al 2007 (41)	2003–2004		25–64	993	1 district	BMI (height and weight measured using standard calibrated instruments)	≥25.0 kg/m^2^	1.8%
Trinh et al 2009 (31)	2005		25–64	908	HCMC	BMI (height and weight measured using standard calibrated instruments)	≥25.0 kg/m^2^	15.1%
Trinh et al 2009 (31)	2005		25–64	908	HCMC	BMI (height and weight measured using standard calibrated instruments)	≥23.0 kg/m^2^	33.5%
**Overweight or obesity, female**								
Ta et al 2010 (45)	Not reported		30–70	1,421	HCMC	BMI (height and weight measured using standard calibrated instruments)	≥25.0 kg/m^2^	26.1%
Ahmed et al 2009 (42)	2005		25–64	4,194	Fila Bavi district	BMI (height and weight measured using standard calibrated instruments)	≥25.0 kg/m^2^	1.9%
Ahmed et al 2009 (42)	2005		25–64	4,194	Chililab district	BMI (height and weight measured using standard calibrated instruments)	≥25.0 kg/m^2^	5.9%
Pham et al 2009 (33)	2005		25–64	1,066	Can Tho (southern VN)	BMI (height and weight measured using standard calibrated instruments)	≥25.0 kg/m^2^	14.1%
Tuan et al 2008 (46)	2002		18–65	158,019	National	BMI (height and weight measured using standard calibrated instruments)	≥25.0 kg/m^2^	6.2%
Van Minh et al 2007 (39)	2005		25–64	997	Fila Bavi district	BMI (height and weight measured using standard calibrated instruments)	≥25.0 kg/m^2^	4.0%
Cuong et al 2007 (29)	2004		20–60	771	HCMC	BMI (height and weight measured using standard calibrated instruments)	≥23.0 kg/m^2^	34.0%
Nguyen et al 2007 (47)	2002		19+	50,402	National	BMI (height and weight measured using standard calibrated instruments)	≥25.0 kg/m^2^	6.6%
Son et al 2012 (30)	2002–2008		≥25	5,970	National	BMI (height and weight measured using standard calibrated instruments)	≥23.0 kg/m^2^	22.7%
Tesfaye et al 2007 (41)	2003–2004		25–64	1,023	1 district	BMI (height and weight measured using standard calibrated instruments)	≥25.0 kg/m^2^	1.9%
Trinh et al 2009 (31)	2005		25–64	1,063	HCMC	BMI (height and weight measured using standard calibrated instruments)	≥25.0 kg/m^2^	16.3%
Trinh et al 2009 (31)	2005		25–64	1,063	HCMC	BMI (height and weight measured using standard calibrated instruments)	≥23.0 kg/m^2^	33.0%
**Physical inactivity, male**								
Trinh et al 2010 (28)	2005		25–64	908	HCMC	GPAQ	Light (no or some activity that is less than high and moderate categories)	46.0%
Ahmed et al 2009 (42)	2005		25–64	4,194	Fila Bavi district	GPAQ	Low level of physical activity	63.0%
Ahmed et al 2009 (42)	2005		25–64	4,194	Chililab district	GPAQ	Low level of physical activity	15.0%
Ng et al 2009 (40)	2005		25–64	4,194	Fila Bavi district	GPAQ	No vigorous activity	53.0%
Ng et al 2009 (40)	2005		25–64	4,194	Chililab district	GPAQ	No vigorous activity	42.0%
Pham et al 2009 (33)	2005		25–64	910	Can Tho (southern VN)	Questionnaire (self-report)	Low level (<600 MET-min of moderate and/or vigorous activity/wk)	33.0%
Nguyen et al 2012 (36)	2009		≥25	785	2 provinces	Questionnaire (self-report)	<3,000 MET- min/wk	20.3%
**Physical inactivity, female**								
Trinh et al 2010 (28)	2005		25–64	1,063	HCMC	GPAQ	Light (no or some activity that is less than high and moderate categories)	41.0%
Ahmed et al 2009 (42)	2005		25–64	4,194	Fila Bavi district	GPAQ	Low level of physical activity	53.0%
Ahmed et al 2009 (42)	2005		25–64	4,194	Chililab district	GPAQ	Low level of physical activity	11.0%
Ng et al 2009 (40)	2005		25–64	4,194	Fila Bavi district	GPAQ	No vigorous activity	56.0%
Ng et al 2009 (40)	2005		25–64	4,194	Chililab district	GPAQ	No vigorous activity	68.0%
Pham et al 2009 (33)	2005		25–64	1,066	Can Tho (southern VN)	Questionnaire (self-report)	Low level (<600 MET-min of moderate and/or vigorous activity/wk)	40.0%
Nguyen et al 2012 (36)	2009		25+	1,345	2 provinces	Questionnaire (self-report)	<3,000 MET min/wk	19.3%
**Tobacco use, male**								
Trinh et al 2010 (28)	2005		25–64	908	HCMC	Questionnaire (self-report)	Current smoker	58.0%
Ahmed et al 2009 (42)	2005		25–64	4,194	Fila Bavi district	Questionnaire (self-report)	Current smoker	60.0%
Ahmed et al 2009 (42)	2005		25–64	4,194	Chililab district	Questionnaire (self-report)	Current smoker	52.0%
Pham et al 2009 (33)	2005		25–64	910	Can Tho (southern VN)	Questionnaire (self-report)	Current smoker	68.0%
Van Minh et al 2008 (34)	2005		25–74	1,216	Fila Bavi district	Questionnaire (self-report)	Current smoker	59.0%
Van Minh et al 2007 (39)	2005		25–64	987	Fila Bavi district	Questionnaire (self-report)	Current smoker	63.0%
Cuong et al 2007 (29)	2004		20–60	717	HCMC	Questionnaire (self-report)	Current smoker	62.0%
Ng et al 2006 (40)	2002		25–64	997	Fila Bavi district	Questionnaire (self-report)	Current smoker	57.0%
Giang et al 2008 (35)	2004		18–60	1,695	1 rural district	Questionnaire (self-report)	Current smoker	65.1%
Nguyen et al 2012 (36)	2009		≥25	785	2 provinces	Questionnaire (self-report)	Current smoker	58.8%
Palipudi et al 2012 (49)	2009		15+	4,823	National	Questionnaire (self-report)	Current smoker or user of any tobacco product, either daily or occasionally	47.6%
**Tobacco use, female**								
Trinh et al 2010 (28)	2005	25–64		1,063	HCMC	Questionnaire (self-report)	Current smoker	1.6%
Ahmed et al 2009 (42)	2005	25–64		4,194	Fila Bavi district	Questionnaire (self-report)	Current smoker	0.5%
Ahmed et al 2009 (42)	2005	25–64		4,194	Chililab district	Questionnaire (self-report)	Current smoker	0.4%
Pham et al 2009 (33)	2005	25–64		1,066	Can Tho (southern VN)	Questionnaire (self-report)	Current smoker	1.1%
Van Minh et al 2008 (34)	2005	25–74		1,268	Fila Bavi district	Questionnaire (self-report)	Current smoker	0.7%
Van Minh et al 2007 (39)	2005	25–64		997	Fila Bavi district	Questionnaire (self-report)	Current smoker	0.6%
Cuong et al 2007 (29)	2004	20–60		771	HCMC	Questionnaire (self-report)	Current smoker	1.4%
Ng et al 2006 (40)	2002	25–64		999	Fila Bavi district	Questionnaire (self-report)	Current smoker	0.1%
Giang et al 2008 (35)	2004	18–60		1,728	1 rural district	Questionnaire (self-report)	Current smoker	0.2%
Nguyen et al 2012 (36)	2009	≥25		1,345	2 provinces	Questionnaire (self-report)	Current smoker	3.8%
Palipudi et al 2012 (49)	2009	15+		5,102	National	Questionnaire (self-report)	Current smoker or user of any tobacco product, either daily or occasionally	3.6%

Abbreviations: HCMC, Ho Chi Minh City; VN, Viet Nam; AUDIT, Alcohol Use Disorder Identification Test; OGTT, oral glucose tolerance test; SBP, systolic blood pressure; DBP, diastolic blood pressure; BMI, body mass index; GPAQ, Global Physical Activity Questionnaire; MET, metabolic equivalent.

a Definition refers to the numerical aspect of the definition of the risk factor. Measurement method is also an important part of the definition.

b Using cholesterol-lowering medications and/or having 1 or more of the following: total cholesterol ≥5.17 mmol/L; high-density lipoprotein cholesterol <1.03 mmol/L; low-density lipoprotein cholesterol ≥3.36 mmol/L; triglycerides ≥1.7 mmol/L.

c Fasting blood glucose ≥7.0 mmol/L and/or 2 h after OGTT blood glucose ≥11.1 mmol/L and/or self-reported as currently taking any diabetes medication.

Tobacco use was the most common behavioral risk factor studied (n = 10), followed by alcohol use (n = 6), physical inactivity (n = 5), low fruit and vegetable consumption (n = 3), and high salt intake (n = 1). For physical measurements, being overweight or obese was most commonly studied (n = 14), followed by high blood pressure (n = 11), and abdominal adiposity (n = 4). For biological measurements, 5 studies assessed elevated blood glucose and 3 studies examined elevated cholesterol.

The definition of many of the risk factors varied among studies. Tobacco use was the most consistently defined (as being a current smoker), and prevalence ranged from 47.6% to 68.0% in men, and 0.1% to 3.8% in women. Examining trends over time suggests that prevalence may be increasing in women.

The definition of at-risk alcohol use varied across studies. Prevalence ranged from 9.1% to 61.0% in men, and 0.3% to 5.0% in women. Having fewer than 5 servings of fruit and vegetables per day ranged from 59.4% to 87.0% in men, and 52.0% to 88.0% in women. High salt intake was examined in 1 study; the prevalence of people who preferred daily foods that contained more salt than similar foods ordered by other adult members in the family or people around them was 32.2% in men and 27.1% in women. The definition of physical inactivity also varied widely among studies. Prevalence ranged from 15.0% to 63.0% in men, and 11.0% to 68.0% in women.

For the definition of being overweight or obese, some studies used the standard Western criteria of body mass index at or greater than 25.0 kg/m^2^; others used the Asian criteria of 23.0 kg/m^2^ or greater, and others used both. Using the Asian criteria, the prevalence of overweight or obesity ranged from 18.4% to 33.5% in men, and 22.7% to 34.0% in women.Using the Western criteria, the prevalence of overweight or obesity ranged from 1.8% to 21.7% in men, and 1.9% to 26.1% in women.

Most studies defined high blood pressure as 140/90 mm Hg or higher. In these studies, the prevalence of high blood pressure varied from 11.3% to 31.2% in men, and 8.9% to 25.0% in women. People’s awareness of their high blood pressure may be low in Viet Nam; in 1 study, which asked people whether they had ever been told by a health care worker that they had high blood pressure, only 6% of males and 5% of females answered yes (13). Elevated blood glucose (≥6.1 mmol/L) was less common than other risk factors; prevalence ranged from 1.0% to 9.8%. Elevated total cholesterol (≥5.2 mmol/L) was more common; prevalence ranged from 14.5% to 21.0%.

## Discussion

To our knowledge, this is the first literature review of chronic disease risk factors in Viet Nam. Our review indicates that a substantial amount of information has been gathered and re-emphasizes the extent of these risk factors and the likely influence they are having on the rapidly growing burden of chronic diseases in Viet Nam. In particular, it demonstrates the extent of tobacco and alcohol use among men, the high proportion of diets that do not meet international criteria for fruit and vegetable consumption, the growing prevalence of overweight and obesity, the low levels of physical activity, and the high levels of hypertension and hypercholesterolemia.

Most chronic diseases can be prevented and controlled. Affordable solutions exist to reduce the level of exposure of individuals and populations to the common modifiable risk factors, to improve access to health care, and to prevent complications and disability in those with established noncommunicable diseases (5). Although many international initiatives target chronic diseases (14–18), policies and programs for the prevention and management of these diseases in much of Asia are in their infancy (19). In many Asian countries, chronic diseases do not receive the resources necessary for the development and implementation of policies and programs because of the more established needs of infectious disease control (19).

At the recent United Nations General Assembly High-level Meeting on Non-Communicable Diseases, Secretary General Ban Ki-moon emphasized that public policy makers in low- and middle-income countries are increasingly challenged to establish effective programs to prevent and control chronic diseases. Consequently, many have requested technical support to scale up efforts and build sustainable institutional capacity; however, these requests remain largely unanswered. Ban Ki-moon concluded, “If the high mortality and socioeconomic impact experienced by low- and middle-income countries are to be reduced, global development initiatives must take into account the prevention and control of such diseases as an integral part of their priorities” (5). Successful programs for the prevention of chronic diseases are based on a comprehensive understanding of the context-specific risk factors of these diseases (19).

Our study has limitations. The main weakness of the information available for Viet Nam is that much of the data are least 7 years old, and all are at least 3 years old. The demographic and epidemiological profile in Viet Nam continues to change. Chronic diseases are causing an increasing burden in terms of mortality and morbidity (6,7). More recent data would be useful to assess the prevalence and predictors of the most common risk factors for chronic diseases in Viet Nam as well as to track their trends over time.

Furthermore, data collected should be more specific to the chronic disease pattern that has emerged, such as estimating total caloric and salt intake, both of which are risk factors for stroke and hypertension (20). Such estimates will require methods adapted to the Vietnamese population since the validity of dietary assessment methods varies across population groups (21). In addition, musculoskeletal conditions cause a large burden in low- and middle-income countries (3,22–27), and information on risk factors for these conditions should be included in initiatives such as WHO STEPS. There is also a need to collect information on community knowledge, attitudes, and perceptions of chronic disease in Viet Nam, both in terms of the influence of risk factors and their consequences on health, and the socioeconomic aspects of health care provision. Finally, from a health systems perspective, there is a need to assess the nature and availability of primary health care services for treating chronic disease in Viet Nam to design appropriate interventions to address the growing burden from these conditions.

Most of the studies we reviewed had sample sizes of approximately 2,000; while this size may provide adequate statistical power for performing some age- and sex-stratified analyses at the national level, it may not be adeaquate for performing subnational analyses. Also, the fact that the definition of many of the risk factors varied across studies is important to recognize when comparing results. Many studies assessed prevalence of risk factors but failed to undertake a more in-depth analysis of the exposures, including other risk factors and social determinants of health, that may lead to chronic disease risk factors, and the outcomes, including morbidity and mortality, that may result from these chronic disease risk factors. This study indicates that risk factors for chronic diseases are common in Viet Nam. Although there is existing information on these risk factors, more recent and context-specific information is required for planning and monitoring interventions against risk factors and chronic disease in Viet Nam.
